# Two-Step Method for Integrating Triply Periodic Minimal Surface Structures of Different Types with Large Differences in Pore Size

**DOI:** 10.3390/ma19214596

**Published:** 2026-10-30

**Authors:** Nan Yang, Yongyi Huang, Wanyu Hu, Aoran Feng

**Affiliations:** Intelligent Manufacturing Key Laboratory of Ministry of Education, College of Engineering, Shantou University, Shantou 515063, China; 24yyhuang1@stu.edu.cn (Y.H.); 25wyhu@stu.edu.cn (W.H.); 25arfeng@stu.edu.cn (A.F.)

**Keywords:** TPMS structures, two-step method, gradient porous structure, additive manufacturing, structural stiffness

## Abstract

Aiming at the integration of triply periodic minimal surface (TPMS) structures with different types and large differences in pore size, a two-step transition method is proposed. Taking P-type and G-type TPMSs as examples, the unit cell number of the P-structure is gradually adjusted to the target size first, and then a smooth transition between P- and G-structures with the same unit number is realized via a Cauchy linear weight function, eliminating geometric defects caused by direct hybridization. Samples are modeled by Mathematica 11.2 and fabricated via selective laser sintering of nylon powder. The experimental results show that the transition zone is defect-free, the volume fraction is close to the designed value, and the structural stiffness is increased by ~40% compared with the traditional direct transition method. This work provides an effective strategy for designing and manufacturing high-performance gradient porous structures via additive manufacturing.

## 1. Introduction

Triply periodic minimal surface (TPMS) porous lattices are a class of surface-based cellular architectures characterized by zero mean curvature, continuous surface morphology, highly controllable pore geometry and outstanding multi-functional performance. Benefiting from these intrinsic merits, TPMS structures have attracted tremendous research interest across additive manufacturing, biomedical implant engineering, lightweight structural design and energy-absorption component development over the past decade [1,2,3,4,5]. For biomedical scenarios, TPMS scaffolds can mimic the elastic modulus and permeability of human bone tissues to facilitate cell ingrowth. In engineering fields, TPMS lattices deliver high specific stiffness and energy-absorption capability under static and dynamic loading conditions.

Functionally graded TPMS components, whose pore topology or pore size varies spatially, further extend the application boundary of porous materials. In many practical engineering requirements, a single component is expected to integrate multiple topological features: for instance, one region requires P-type TPMS, while another region demands G-type TPMS, accompanied by a large discrepancy in pore dimensions between these two regions [6,7,8]. Under such circumstances, integrating different types of TPMS structures with large pore-size differences becomes an urgent technical requirement for fabricating high-performance functional-gradient porous parts.

Nevertheless, conventional direct integration strategies still face prominent technical bottlenecks when handling a cross-type TPMS transition with large pore-size gaps. Most existing methods adopt a single weight-based transition function to directly blend the implicit equations of distinct TPMS unit cells with different pore sizes. This direct mathematical interpolation frequently triggers topological mutations, disconnected solid fragments and irregular distorted geometry within the transition zone [9,10]. These undesired geometric anomalies cannot be reliably reproduced during additive manufacturing. A large number of tiny disconnected features will be removed during post-processing, which yields obvious internal defects, reduces the actual volume fraction, and degrades the elastic modulus and structural stiffness of the final components [11,12]. Consequently, the mechanical reliability and service performance of gradient porous structures are severely compromised.

Current published studies can be roughly divided into two streams, and both exhibit evident limitations for our target scenario. The first stream focuses on gradient regulation within a single TPMS topology. These well-established approaches realize continuous adjustment of pore size or porosity while keeping the unit-cell type unchanged [13,14,15]. Such single-type gradient algorithms are computationally stable and easy to implement, but they cannot switch topological categories. Therefore, they fail to satisfy multi-functional coupling demands that need the advantages from multiple TPMS topologies simultaneously. The second stream explores cross-type TPMS hybridization. A small number of existing works have attempted to realize morphology transitions between different TPMS types [16,17,18]. However, these approaches mostly work well only for cases with close pore sizes. Once the pore-size difference becomes considerable, direct hybridization produces severe structural incompatibility and frequently leads to manufacturing failure. To sum up, few available design frameworks can simultaneously achieve two critical targets: cross-topology integration and stable transition under large pore-size gradients.

To address the above-mentioned technical challenges, this paper proposes a two-step integration method for heterogeneous TPMS structures with large pore-size differences. An intermediate structure with uniform unit-cell dimension is introduced, and the whole gradient transition process is separated into two sequential stages: same-topology pore-size grading and cross-topology smooth hybridization. This design framework avoids abrupt topological change and transition-zone geometric defects, preserves the designed volume fraction, and maintains excellent structural stiffness for integrated heterogeneous TPMS lattices. This work delivers a feasible design route for high-performance gradient porous structures and expands the application scope of TPMS-based additive-manufactured components.

## 2. Methods

### 2.1. Structural Design

Here, we introduce a two-step method for integrating a graded structure with different types and a large difference in pore sizes. P-type and G-type TPMS structures are utilized as examples. We intend to generate a graded structure from P-structure with *m* × *m* units (P*_m_*) to G-structure with *n* × *n* units (G*_n_*) (*m* < *n*). As G-structure has a good connection to P-structure with the same size units (P*_n_*), we use P*_n_* as the intermediate structure. Thus, this method has two steps: (1) P*_m_* → P*_n_*; (2) P*_n_* → G*_n_*.

(1) P*_m_* → P*_n_*:

The P*_m_* structure can be expressed as

(1) u(x,y,z)=cos(a x)+cos(a y)+cos(a z)−0.6≥0

where *a* = *m π* in the −1 ≤ *x* ≤ 1 and −1 ≤ *y* ≤ 1 and −1 ≤ *z* ≤ 1 domains.

The graded structure P*_m_* → P*_n_* can be generated by

(2) u(T(z) x,T(z) y,z)≥0

where *T*(*z*) is the transition function in the transition region_1_ −*h* ≤ *z* ≤ *h*:

(3) $$T(z)=\frac{2h}{(b+1)h+(b-1)z}$$

where the transition coefficient is *b* = *m*/*n*.

(2) P*_n_* → G*_n_*:

The G*_n_* structure can be expressed as

(4) v(x,y,z)=cos(a x)sin(a y)+cos(a y)sin(a z)+cos(a z)sin(a x)−0.5≥0

where *a* = *n π* in the −1 ≤ *x* ≤ 1 and −1 ≤ *y* ≤ 1 and −1 ≤ *z* ≤ 1 domains.

The graded structure P*_n_* → G*_n_* can be generated by

(5) $$f\left(x,y,z\right)=W_{t}\left(z\right)\cdot u\left(x,y,z\right){|}_{a=n\;\pi }+\left(1-W_{t}\left(z\right)\right)\cdot v\left(x,y,z\right){|}_{a=n\;\pi }\geq 0$$

where *W_t_*(*z*) is the Cauchy linear weight function in the transition region_2_ *h* ≤ *z* ≤ 3*h*:

(6) $$W_{t}\left(z\right)=-\frac{z-3h}{2h}$$

**Figure 1:**
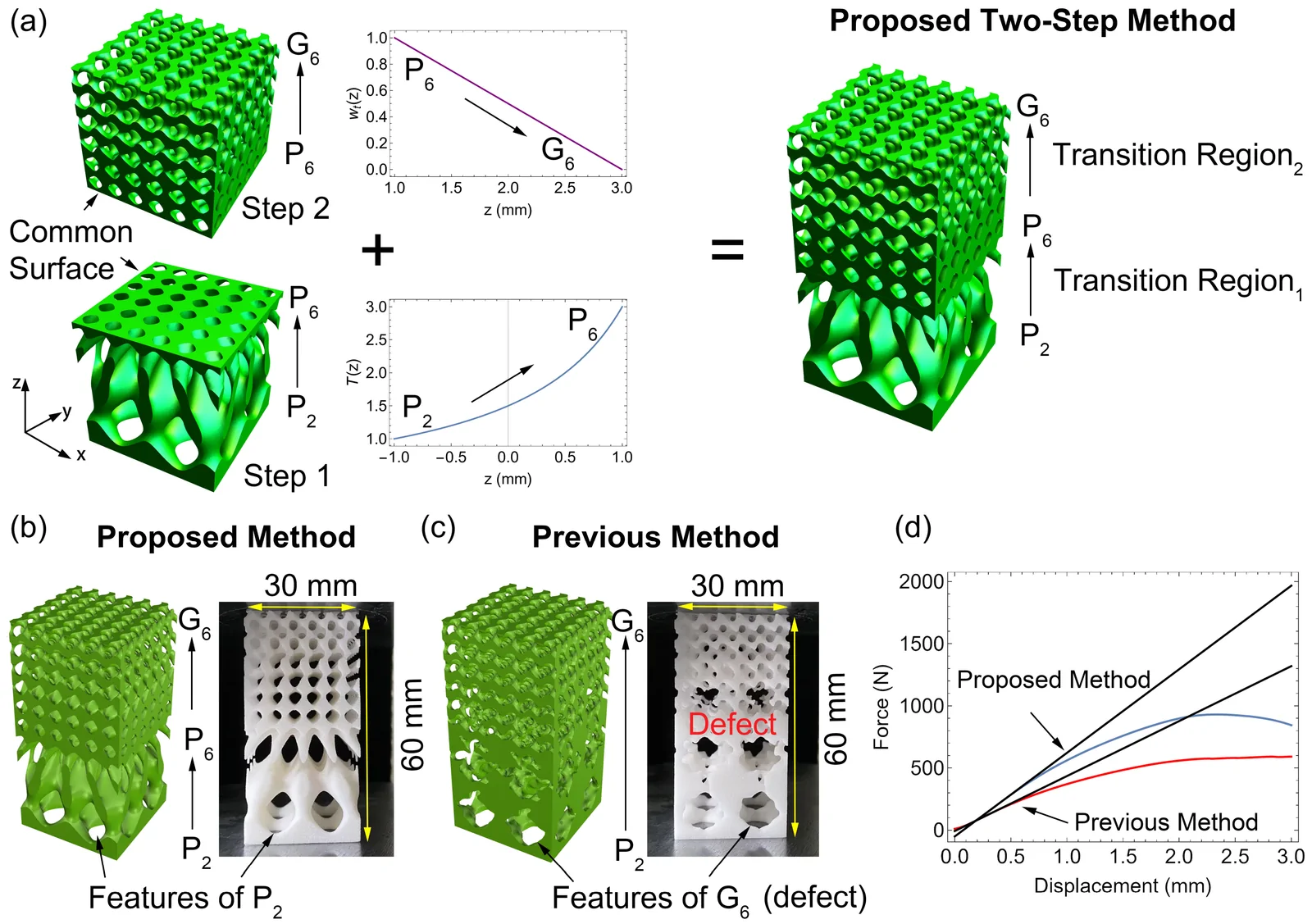
Structural design, fabrication and quasi-static compression test. (**a**) Schematic illustration of the proposed two-step strategy for constructing graded P_2_ →P_6_ →G_6_ structure; the sub-figures show the two individual gradient stages, transition–function curves and assembly logic. (**b**) CAD model and SLS-fabricated physical sample from the proposed two-step method, showing well-preserved P_2_ features without obvious defects. (**c**) CAD model and physical sample from the conventional direct-transition method, where severe geometric defects can be observed within the transition zone. (**d**) Force–displacement curves obtained from quasi-static compression tests for both groups, used to compare structural stiffness (blue curve: proposed method, red curve: previous method).

Finally, the substructures P*_m_* → P*_n_* and P*_n_* → G*_n_* are assembled with the common surface P*_n_* (see Figure 1a). All structures are dimensionless and then scaled to the target physical size.

### 2.2. Sample Fabrication

3D STL models were constructed in Mathematica 11.2 by applying the design method presented in Section 2.1. Selective laser sintering (SLS, brand: pFormics Plus 403P) with nylon powder (FS3300PA) was employed for sample fabrication, with a powder density of 0.48 g/cm^3^, particle diameter ranging from 10 to 50 μm, and a fabricated solid density of 0.97 g/cm^3^. A 60 W laser with a spot diameter of 70 μm was utilized during the sintering process with a layer thickness of 0.1 mm, a laser scan speed of 7.6 m/s, and a hatch spacing of 0.25 mm. Afterward, residual powder was cleaned away, and sandblasting was conducted to remove incompletely sintered powder from the sample surfaces. The size of each printed sample was 30 mm × 30 mm × 60 mm. The fabrication method was the same for both the previous and new design methods.

### 2.3. Measurement of Volume Fraction

The solid lattice volume (*V_n_*) of each specimen was determined using the relation *V_n_* = *M_n_*/*ρ_n_*, in which *M_n_* represents the specimen mass, and *ρ_n_* is the density of the parent material (*ρ_n_* = 0.97 g/cm^3^). The volume fraction was then calculated as the quotient between *V_n_* and the outer-envelope cubic volume of the specimen (*V_solid_*), i.e., *V_n_*/*V_solid_*. Here, *V_solid_* denotes the total outer-envelope cubic volume of the printed specimen. All samples had identical outer dimensions of 30 mm × 30 mm × 60 mm, so *V_solid_* = 30 × 30 × 60 mm^3^.

### 2.4. Quasi-Static Compression Test

A CMT5105 universal testing machine (Shenzhen, China; compressive load F ≤ 85 kN, speed: 5 mm/min) was used to perform compression tests on the top surfaces of the specimens. The testing machine applied a vertical displacement of ∆*L* = 3 mm, which was adequate to fully characterize their elasticity relative to their overall height *L* = 60 mm, resulting in a nominal compressive strain of ∆*L*/*L* = 0.05. Three specimens were tested for each design, with the compressive force and corresponding displacement data recorded simultaneously. Structural stiffness was subsequently determined by fitting the force–displacement curves within their elastic deformation ranges. Three replicate specimens were prepared and tested for each design group (previous and new design methods), giving a total of six specimens for quasi-static compression tests. Volume fraction measurement was performed on these same specimens first.

## 3. Results

As an example, we intend to generate a graded structure from P-structure with 2 × 2 units (P_2_) to G-structure with 6 × 6 units (G_6_) with two steps: (1) P_2_ → P_6_ and (2) P_6_ → G_6_. For step 1, we have *a* = 2*π* for the P_2_ structure and utilize Equations (1)–(3) with *b* = *m*/*n* = 1/3, where *T*(*z*) realizes the process P_2_ → P_6_ (see the blue curve in Figure 1a). For step 2, we have *a* = 6*π* for both the P_6_ and G_6_ structures and apply Equations (4)–(6), where *W_t_*(*z*) realizes the process P_6_ → G_6_ (see the purple curve in Figure 1a). For the two steps, *h* = 1. The resulting P_2_ → P_6_ → G_6_ graded structure is shown on the right side of Figure 1a, and a fabricated sample is shown in Figure 1b.

In contrast, the previous method provides a direct transition P_2_ → G_6_ by using a sigmoid function [9,17,18]

(7) $$s\left(z\right)\cdot u\left(x,y,z\right){|}_{a=2\pi }+\left(1-s\left(z\right)\right)\cdot v\left(x,y,z\right){|}_{a=6\pi }\geq 0$$

in the −1 ≤ *x* ≤ 1 and −1 ≤ *y* ≤ 1 and −2 ≤ *z* ≤ 2 domains, where $s\left(z\right)=\frac{1}{1+e^{z}}$. The resulting graded structure is shown in Figure 1c.

Comparing Figure 1b,c, although both have a 30 mm × 30 mm × 60 mm structure, their transition mechanisms are different. For the previous method (Figure 1c), the transition is direct from P_2_ to G_6_, and there is a defect zone in the middle. The functions’ abnormal morphology from direct interpolation in the hybridization region resulted in many isolated and small geometric shapes, which were removed during the manufacturing process, leading to a low manufacturing volume fraction of 0.27 ± 0.01. At the bottom of the sample, the P_2_ structure already exhibits features of the G_6_ structure, which indicates the inaccuracy of geometric modeling.

On the contrary, in the proposed method (Figure 1b), the transition region is defect-free, and the manufacturing volume fraction is 0.32 ± 0.005, which is close to the designed 0.33. Additionally, the P_2_ features at the bottom are distinct. Further, the quasi-static compression test (Figure 1d) shows that the stiffness of the samples (the slope of the force–displacement curve in the elastic region) constructed using the proposed method is 655.1 ± 17.4 N/mm, which is increased by ~40% compared with the previous method (465.7 ± 23.5 N/mm).

Consistent with previous reports on direct TPMS hybridization [17,18], our experimental observations confirm that conventional single-weight-function blending produces severe geometric defects when the pore sizes differ substantially. Such artifacts cannot survive additive-manufacturing post-processing, leading to substantial deviation between realized and designed volume fraction and deteriorated mechanical stiffness. Most existing gradient TPMS design strategies are limited to pore-size tuning within one single topology [13,14,15], which cannot realize topology switching between different types of lattices. A small number of cross-type transition approaches have been proposed, yet they perform reliably only for cases with minor unit-cell size mismatch [9]. By contrast, our two-step workflow introduces intermediate equal-cell-size structures to separate size grading and topological hybridization. Consequently, the transition zone remains defect-free, the volume fraction closely matches the target value, and the structural stiffness rises by approximately 40% relative to the direct-transition benchmark. This demonstrates the effectiveness of unit-size homogenization for handling large pore-size differences in heterogeneous TPMS integration.

## 4. Conclusions

This work proposes a two-step integration strategy for heterogeneous TPMS lattices with large pore-size differences. By introducing an intermediate TPMS structure of uniform unit-cell size, the cross-type gradient transition is split into same-type size grading and equal-cell topological hybridization, avoiding topological mutation and transition-region geometric defects found in conventional direct-hybridization methods.

Taking P-type and G-type TPMS as examples, SLS-manufactured nylon samples demonstrate a nearly defect-free transition zone. The measured volume fraction (0.32 ± 0.005) closely matches the designed value of 0.33. Compared with the traditional direct-transition design, the structural stiffness increases by approximately 40%.

This two-step design exhibits good robustness for constructing cross-type gradient TPMS structures. It provides a practical technical route for additively manufactured porous components for biomedical implants, energy-absorption and lightweight applications.

## Data Availability

The original contributions presented in this study are included in the article. Further inquiries can be directed to the corresponding author.
